# 1404. Factors Associated to Decrease in Quality of Life After Mild-Moderate COVID-19: A Cross-Sectional Study.

**DOI:** 10.1093/ofid/ofac492.1233

**Published:** 2022-12-15

**Authors:** Alberto Ordinola Navarro, Hector Rivera Villegas, Bruno Ali Lopez Luis

**Affiliations:** Instituto Nacional de Ciencias Médicas y Nutrición Salvador Zubirán, Mexico, Distrito Federal, Mexico; Instituto Nacional de Ciencias Médicas y Nutrición Salvador Zubirán, Mexico, Distrito Federal, Mexico; Centro Médico Nacional 20 de Noviembre, Mexico, Distrito Federal, Mexico

## Abstract

**Background:**

Post-COVID-19 alterations have been recognized even after mild disease. We aimed to assess which factors are the main contributors to a decrease in quality of life(QOL) of patients with different times elapsed from the COVID-19 diagnosis.

**Methods:**

A cross-sectional study from January 2021 to April 2021 in a Referral Center in Mexico City.

Patients were invited for a follow-up visit in which a structured questionnaire about symptoms, the EQ-5D-5L QOL for QOL, and an objective olfactory evaluation with The Sniffin’ Sticks Screening 12 test.

**Results:**

We included 179 patients, 64% were female with a median age of 33 years. The median time since COVID-19 diagnosis until the evaluation was 219 days (IQR, 94-255). Persistent symptoms were present up to in 158/179 (88%), fatigue, pain/discomfort and cognitive alterations were present in 61%, the median EQ-5D-5L index value preCOVID-19 was 1 (IQR, 0.94-1) and post- COVID-19 was 0.87 (IQR, 0.80-0.94), *P*< 0.001. There were 101/179 (56%) patients with decreased QOL; In the multivariate analysis, post-COVID-19 pain (aOR, 2.5; *P*= 0.01), anxiety (aOR, 13; *P*= 0.03), and the persistence of three or more symptoms (aOR, 2.6; *P*= 0.05) were factors associated with decreased QOL.

**Figure:**
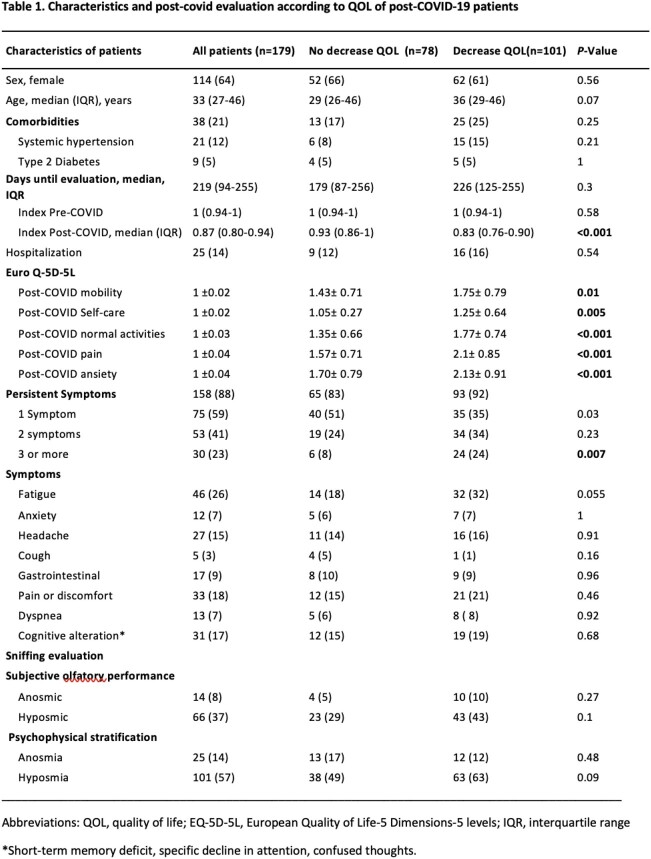

**Figure:**
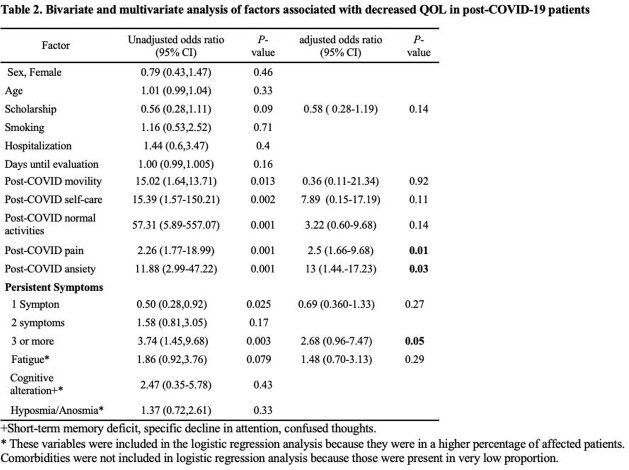

**Figure:**
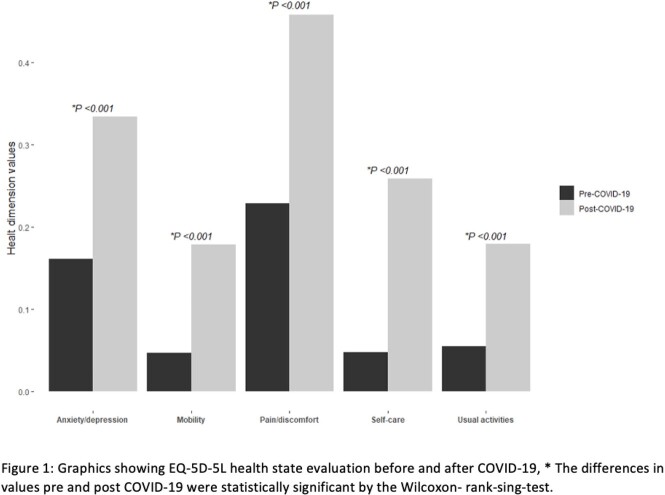

**Conclusion:**

Within the post- COVID-19 alterations, psychological and physical factors such as Pain/discomfort, anxiety, and persistent symptoms explained the decreased QOL in the post-COVID-19 patient. These alterations were present as early as 30 days to more than eight months.

**Disclosures:**

**All Authors**: No reported disclosures.

